# Posterior capsular release is a biomechanically safe procedure to perform in total knee arthroplasty

**DOI:** 10.1007/s00167-018-5094-0

**Published:** 2018-08-09

**Authors:** K. K. Athwal, P. E. Milner, G. Bellier, Andrew A. Amis

**Affiliations:** 1 Department of Mechanical Engineering, Imperial College London, Exhibition Road, London, SW7 2AZ UK; 2 Cabinet Goethe, 23 Avenue Niel, Paris, 75017 France; 3 Musculoskeletal Surgery Group, Department of Surgery and Cancer, Imperial College London School of Medicine, Charing Cross Hospital, W6 8RF London, UK

**Keywords:** Knee replacement, Posterior capsule release, Stability, Total knee arthroplasty

## Abstract

**Purpose:**

Surgeons may attempt to strip the posterior capsule from its femoral attachment to overcome flexion contracture in total knee arthroplasty (TKA); however, it is unclear if this impacts anterior–posterior (AP) laxity of the implanted knee. The aim of the study was to investigate the effect of posterior capsular release on AP laxity in TKA, and compare this to the restraint from the posterior cruciate ligament (PCL).

**Methods:**

Eight cadaveric knees were mounted in a six degree of freedom testing rig and tested at 0°, 30°, 60° and 90° flexion with ± 150 N AP force, with and without a 710 N axial compressive load. After the native knee was tested, a deep dished cruciate-retaining TKA was implanted and the tests were repeated. The PCL was then cut, followed by releasing the posterior capsule using a curved osteotome.

**Results:**

With 0 N axial load applied, cutting the PCL as well as releasing the posterior capsule significantly increased posterior laxity compared to the native knee at all flexion angles, and CR TKA states at 30°, 60° and 90° (*p* < 0.05). However, no significant increase in laxity was found between cutting the PCL and subsequent PostCap release (n.s.). In anterior drawer, there was a significant increase of 1.4 mm between cutting the PCL and PostCap release at 0°, but not at any other flexion angles (*p* = 0.021). When a 710 N axial load was applied, there was no significant difference in anterior or posterior translation across the different knee states (n.s.).

**Conclusions:**

Posterior capsular release only caused a small change in AP laxity compared to cutting the PCL and, therefore, may not be considered detrimental to overall AP stability if performed during TKA surgery.

**Level of evidence:**

Controlled laboratory study.

## Introduction

In total knee arthroplasty (TKA), the knee may be found to be too stiff in extension, causing an extension deficit. Being unable to fully extend the knee requires continuous quadriceps contraction during daily routine movements or standing, leading to tiredness and reduced function [14, 19, 24]. One proposed surgical technique to correct this flexion contracture is resecting additional bone from the distal femur, but that may lead to raising the joint line [20]. Whiteside and Milhalko determined that after removing osteophytes, the primary step should be collateral ligament release and a secondary step being posterior capsular release, with distal femoral cuts only considered if still uncorrected [17, 23]. It was previously believed that releasing the PCL could correct flexion contracture, but this has been found experimentally to increase the flexion gap relative to the extension gap and thus is counterproductive [16]. Alternatively, full extension may be gained by releasing the posterior capsule from its femoral attachment [4, 5, 12, 17]. However, if there was an adverse effect on anterior–posterior (AP) stability by releasing the capsule, then it may be advisable to avoid this and instead recut the femur.

LaPrade et al. [15] described the posterior aspect of the knee with the following anatomy: the semimembranosus muscle with eight distinct soft tissue attachments distal to the main tendon (including a lateral expansion to the oblique popliteal ligament); a posterior capsular thickening which extends from the popliteus musculotendinous junction to the posterior aspect of the intercondylar notch; a popliteofibular ligament and a fabellofibular ligament. However, the biomechanical function of this network of structures is not well understood. It is known, for example, that the oblique popliteal ligament restrains against knee hyperextension [18]; however, its importance to stability particularly in AP  drawer is unknown. Gollehon et al. [9] found the posterolateral arcuate complex to be a secondary posterior stabiliser, but did not investigate the posterior capsule in detail. Furthermore, the meniscofemoral ligaments have been found to be secondary posterior drawer restraints [10]; therefore, it is unclear whether in the TKA setting (when the meniscus is resected) other posterior structures may become more important to stability. Recent robotic studies of TKA stability have investigated both the constraint of the implant and the effects of soft tissue releases [2, 3], but did not examine posterior capsular releases.

The primary aim of the study was to investigate if releasing the posterior capsule in an implanted knee caused a large increase in AP laxity which would, therefore, invalidate the use of the technique in TKA surgery. The null hypothesis was that releasing the posterior capsule would not increase laxity when the PCL had been previously cut.

## Materials and methods

Eight fresh-frozen human cadaveric legs (six male and two female) of mean age 78 (standard deviation ± 10 years) were obtained from a tissue bank (four left-sided and four right-sided). The legs had been disarticulated through the hip, and were MRI and X-ray imaged ready for ‘patient’-specific TKA cutting guides. The knees were separated by cutting 170 mm from the joint line both distally on the tibia/fibula and proximally on the femur. The fibula was fixed to the tibia in an anatomic position with a distal tricortical bone screw. The tibia was then cemented in a 60-mm-diameter cylindrical steel pot with polymethyl-methacrylate (PMMA, Simplex, Kemdent, UK). The joint capsule was opened with a midline skin incision and a medial parapatellar arthrotomy, then a jig with a pointer was used to align the centre of the tibial plateau (between the tips of the tibial spines) with the axis of the bone pot [1]. The femur was cemented using PMMA in a bone pot secured in situ in the testing rig, so that it was aligned with the knee in full extension and the posterior condylar axis parallel to the base of the rig.

### Testing rig

**Fig. 1:**
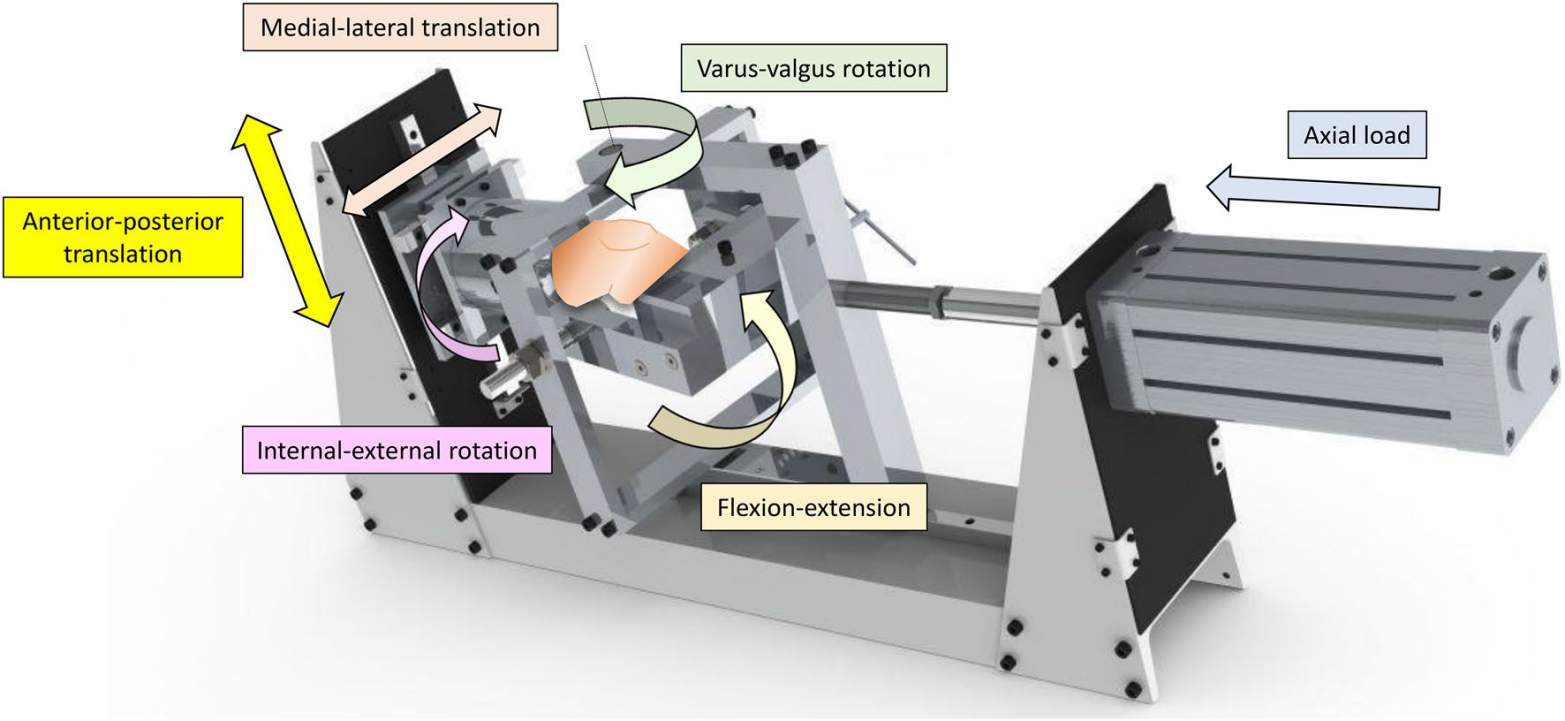
The stability testing rig

A purpose-built rig was designed to be used in conjunction with a materials testing machine (Model 5565, Instron Ltd, High Wycombe, UK). The tibia was mounted in a fixture attached to the moving crosshead of the Instron, whilst the femur was mounted in a pivot frame on linear bearings (Fig. 1). The Instron applied an AP force/displacement to the tibia mounted in the rig at a fixed angle of flexion, whilst the other degrees-of-freedom were unconstrained and free to translate/rotate. The pivot point of the femoral frame could be adjusted medially–laterally to vary the load distribution between the medial and lateral knee compartments; in this study the knees were maintained at a medial:lateral loading distribution of 60:40 throughout testing [25]. To simulate a weight-bearing compressive load on the tibia, a pneumatic cylinder applied a 710 N force in the axial direction [11].

### Test protocol

The native, intact knee with bone pots was mounted into the test rig. The knee was manually flexed 20 times to minimise soft tissue hysteresis, then a ± 150 N AP force was applied when the knee was at full extension, 30°, 60° and 90° flexion. ±150 N was chosen in line with a previously published recommendation on AP laxity testing on cadaveric knees [6]. Three pre-conditioning AP cycles were applied and the resulting AP force versus translation data (directly read from the materials testing machine with accuracy ± 0.1 mm and ± 0.5 N) were collected on the fourth cycle. A previous study demonstrated an intra-rater repeatability of the test rig as a 95% confidence interval of 1 mm [11].

To find the neutral AP position of the knee at each angle, a starting position approximated by eye was chosen, and a ± 3 mm AP draw was applied. The true neutral AP position was then defined at the point of inflection of the force–displacement hysteresis loop, when it was symmetrical above and below the zero force axis.

After the native knee was tested, a deep dished CR TKA (Legion, Smith & Nephew, Memphis, TN, USA) was implanted by an experienced consultant surgeon using a medial parapatellar approach. During pilot studies, it was found that a standard high-flexion CR TKA subluxed anteriorly before reaching 150 N at 60° and 90° and, therefore, it was decided that a more congruent insert would be more appropriate. Femoral and tibial cuts were made using patient-specific guides (Visionaire, Smith & Nephew, Memphis, TN, USA) based on the MRI and X-ray images taken prior to testing. 9.5 mm was resected from the distal femur (referenced from the most distal side of the femur) to account for the thickness of the implant, and 9 mm of bone from the proximal tibia, respectively (referenced from the most superior aspect of the tibial surface) to allow for the thinnest available polyethylene tibial insert (9 mm) to be used. The posterior tibial slope was set at 3 degrees to account for the slope already built into the articular insert. The tibial implant was cemented to the bone, whereas the femoral component was press-fit. This press-fit has previously been shown to give secure fixation at experimental loads [7]. The following stages were sequentially performed and tested at full extension, 30°, 60° and 90° knee flexion with ± 150 N AP force, both with and without 710 N axial loads:

1. The native, intact knee was tested.
2. The CR TKA was implanted with a deep dished insert
3. The PCL was resected.
4. The posterior capsule (PostCap) was released with the knee flexed at 90°. The femoral component was removed for an unobstructed view of the posterior capsule, and then a curved osteotome was used to elevate fibres from the distal femoral cortex behind the condyles. Further release of the medial and lateral fibres of the gastrocnemius was performed with a scalpel. The femoral component was then press fit back on for testing.

### Statistical analysis

Statistical analysis was performed in SPSS 23 (IBM SPSS Statistics, version 22, Armonk, NY). To investigate each of the hypotheses set out in the introduction, multiple two-way repeated-measures analyses of variance (RM ANOVAs) were performed to compare knee laxity to the knee state across different flexion angles. When differences were found between successive knee states, post hoc paired *t* tests with Bonferroni correction were applied at individual flexion angles. Significance level was set at *p* < 0.05. Post hoc power analysis of paired *t* tests indicated that, when comparing laxities with the standard deviations calculated in eight knees, significant changes of 3.2 mm could be detected with 80% power and 95% confidence.

Separate analyses were performed for anterior laxity with no axial load; anterior laxity with 710 N axial load; posterior laxity with no axial load; and posterior laxity with 710 N axial load.

## Results

### Anterior translation

**Fig. 2:**
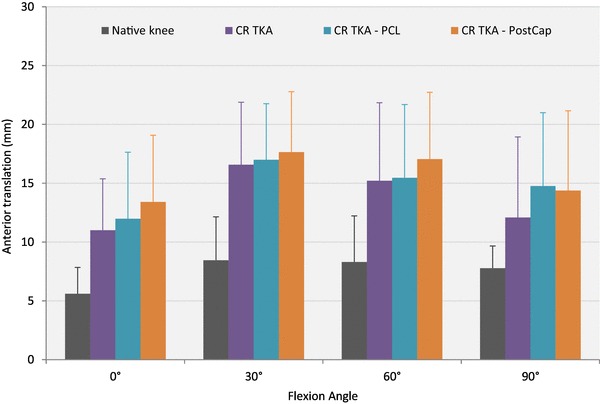
Mean translation in response to a 150 N anterior force with no axial force applied (error bars denote standard deviation). *CR TKA* cruciate-retaining total knee arthroplasty with deep-dished insert, *PCL* posterior cruciate ligament, *PostCap* posterior capsule release

**Fig. 3:**
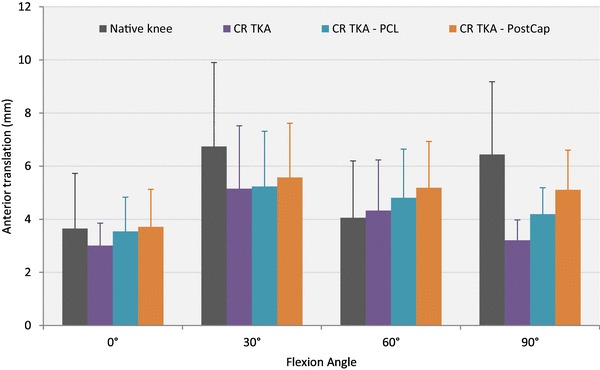
Mean translation in response to a 150 N anterior force with a 710 N axial force applied (error bars denote standard deviation). *CR TKA* cruciate-retaining total knee arthroplasty with deep-dished insert, *PCL* posterior cruciate ligament, *PostCap* posterior capsule release

With 0 N axial load applied (Table 1; Fig. 2), releasing the PostCap significantly increased laxity by 1.4 mm compared with cutting the PCL at 0° (*p* = 0.021). However, when a 710 N axial load was applied, no significant difference was found across the different knee states (Fig. 3).

**Table 1:** Mean anterior translation in mm (with standard deviation), in response to 150 N anterior force with 0 and 710 N axial force applied (*n* = 8)

Flexion angle	Native knee	CR TKA	CR TKA–PCL	CR TKA–PCL–PostCap
0 N axial force				
0°	5.6 (2.2)	11.0 (4.4)	12.0 (5.7)	13.4 (5.7)^γ^
30°	8.5 (3.7)	16.6 (5.3)	17.0 (4.8)	17.7 (5.1)
60°	8.3 (3.9)	15.2 (6.6)	15.5 (6.2)	17.1 (5.7)
90°	7.8 (1.9)	12.1 (6.8)	14.8 (6.2)	14.4 (6.8)
710 N axial force				
0°	3.6 (2.1)	3.0 (0.8)	3.5 (1.3)	3.7 (1.4)
30°	6.7 (3.2)	5.2 (3.4)	5.2 (2.1)	5.6 (2.0)
60°	4.1 (2.1)	4.3 (1.9)	4.8 (1.8)	5.2 (1.7)
90°	6.4 (2.7)	3.2 (0.8)	4.2 (1.0)	5.1 (1.5)

Key to content: *CR TKA* cruciate-retaining total knee arthroplasty with deep-dished insert, *PCL* posterior cruciate ligament, *PostCap* posterior capsule release

^γ^Significant difference from the CRDD TKA–PCL state (*p* < 0.05)

### Posterior translation

**Fig. 4:**
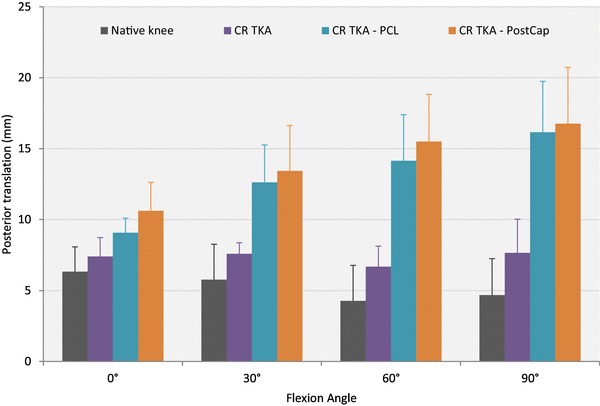
Mean translation in response to a 150 N posterior force with no axial force applied (error bars denote standard deviation). *CR TKA* cruciate-retaining total knee arthroplasty with deep-dished insert, *PCL* posterior cruciate ligament, *PostCap* posterior capsule release

**Fig. 5:**
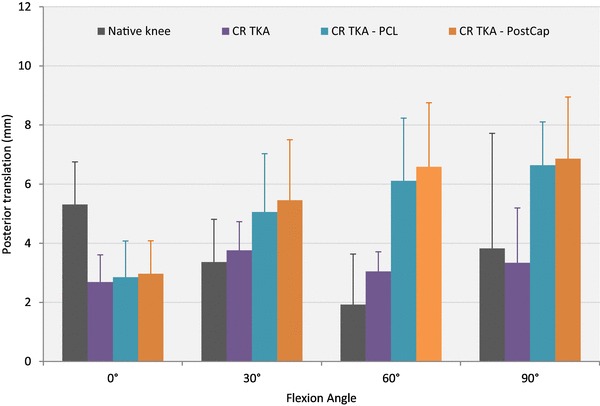
Mean translation in response to a 150 N posterior force with a 710 N axial force applied (error bars denote standard deviation). *CR TKA* cruciate-retaining total knee arthroplasty with deep-dished insert, *PCL* posterior cruciate ligament, *PostCap* posterior capsule release

With 0 N axial load applied (Table 2; Fig. 4), resecting the PCL increased posterior laxity significantly compared to the native knee and CR TKA states at 30°, 60° and 90° (*p* < 0.05). Releasing the PostCap significantly increased posterior laxity compared to the native knee at all flexion angles, and CR TKA states at 30°, 60° and 90° (*p* < 0.05). However, no significant increase in laxity was found between the PCL and PostCap steps. When a 710 N axial load was applied, no significant difference was found across the different knee states (Table 2; Fig. 5).

**Table 2:** Mean posterior translation in mm (with standard deviation), in response to 150 N posterior force with 0 and 710 N axial force applied (*n* = 8)

Flexion angle	Native knee	CR TKA	CR TKA—PCL	CR TKA—PCL–PostCap
0 N axial force				
0°	6.3 (1.7)	7.4 (1.3)	9.1 (1.0)	10.6 (2.0)*
30°	5.8 (2.5)	7.6 (0.8)	12.6 (2.6)*^,β^	13.4 (3.2)*^,β^
60°	4.3 (2.5)	6.7 (1.4)	14.2 (3.2)*^,β^	15.5 (3.3)*^,β^
90°	4.7 (2.6)	7.7 (2.4)	16.2 (3.6)*^,β^	16.8 (4.0)*^,β^
710 N axial force				
0°	5.3 (1.4)	2.7 (0.9)	2.9 (1.2)	3.0 (1.1)
30°	3.4 (1.4)	3.8 (1.0)	5.1 (2.0)	5.5 (2.0)
60°	1.9 (1.7)	3.0 (0.7)	6.1 (2.1)	6.6 (2.2)
90°	3.8 (3.9)	3.3 (1.9)	6.6 (1.5)	6.9 (2.1)

Key to content: *CR TKA* cruciate-retaining total knee arthroplasty with deep-dished insert, *PCL* posterior cruciate ligament, *PostCap* posterior capsule release

*Significant difference from the native state (*p* < 0.05)

^β^Significant difference from the CR TKA state (*p* < 0.05)

## Discussion

The most important finding of the study was that releasing the posterior capsule only caused a small change in AP laxity compared to cutting the PCL when the knee with a CR TKA was non weight-bearing, and did not increase laxity significantly in the loaded knee; therefore, posterior capsular release may not be considered detrimental to overall AP stability if performed during TKA surgery. Given that the posterior cruciate-substituting (PS) TKA is inherently more constrained than the CR TKA, it follows that this finding applies also to PS TKA [8, 13].

The largest effect after posterior capsule release was found at 0° with 1.4 mm increase in anterior laxity with no axial load applied; there was minimal laxity change when 710 N axial body weight was applied. When compared to the 8.5 mm increase in posterior laxity at 90° after cutting the PCL in a deep-dished implant, this laxity change, although statistically significant, is not large in the clinical setting. When comparing stability between the loaded and unloaded experiments, it is clear that stability of TKA is derived by having concave articular surfaces under axial joint compression. For comparison at 0°, the unloaded native knee experienced on average 6 mm anterior drawer and 6 mm posterior drawer, which slightly reduced to 4 and 5 mm, respectively, when loaded. In contrast, the unloaded anterior drawer of the CR state was 11 mm at 0°, which reduced dramatically under applied axial load to 3 mm. The corresponding posterior drawer was 7 mm for unloaded state, reducing to 3 mm when axially loaded.

The role of the posterior aspect of the knee has not been investigated in great detail, and this study is the first to investigate the effect of the posterior capsular release on stability in implanted cadaveric specimens. Morgan et al. investigated the role of different posterior structures and the collateral ligaments in restraining hyperextension in non-implanted cadaveric knees, and found the oblique popliteal ligament to be the primary restraint irrespective of cutting order [18]. With regard to TKA, posterior capsular release has been investigated before in prospective and retrospective clinical trials [12, 17, 23]. Hanratty et al. hypothesised that capsular stripping could improve flexion and range of motion; however, despite finding an immediate increase in knee flexion, no difference was maintained after 3 months or 1 year [12]. Reports of treating flexion contracture post-TKA by posterior capsular release or removal of posterior femoral osteophytes have not considered the possible effect on knee AP stability [14, 21].

A limitation of this study is that cadaveric testing is at time zero. Therefore, healing of the capsule back to the femoral attachment and formation of scar tissue cannot be investigated [12]. However this should not affect the main finding of the study, as healing will only increase stability of the implanted knee post-surgery. Measuring AP laxity with and without a 710 N axial load simulated a comparison between a clinical evaluation of a patient lying supine with relaxed muscles, and a person applying a body weight of 72 kg on the joint; however, this is a simplistic load in direction and magnitude and care should be taken when extrapolating this to kinematics experienced during walking for example.

There is an ongoing debate whether flexion contracture in TKA should be fixed surgically or alternatively treated with continuous physiotherapy postoperatively [14, 22]. This study has found that one such surgical treatment, releasing the posterior capsule from its femoral attachment, did not cause a large detrimental increase in AP laxity at time of surgery. Therefore, posterior capsule release may be considered a safe option to reduce extension deficit. Clinical trials with gait analysis should be performed to highlight how long-term healing may change the effect of posterior capsular release, particularly under full walking loads. Future in vitro studies could quantify how much extension is restored when comparing posterior capsular release to other surgical treatments such as resecting the distal femur, which has a known adverse effect of raising the joint line [20]. The data from the implanted cadavers in this study could also be compared to the constraint of the isolated implants themselves, under the same flexion angles and loading conditions. This would help investigate how much constraint is provided by the different implant geometries compared with the stability provided by the posterior capsule and PCL.

## Conclusion

Releasing the posterior capsule only caused a small change in AP laxity when compared with the increase following TKA or PCL resection and, therefore, may not be considered detrimental to overall AP stability if performed during surgery.
